# Supplementary material for: Molecular Imaging of Ultrasound-Mediated Blood-Brain Barrier Disruption in a Mouse Orthotopic Glioblastoma Model
Source: Pharmaceutics. 2022 Oct 19;14(10):2227. doi: 10.3390/pharmaceutics14102227 (PMC9610067; doi:10.3390/pharmaceutics14102227)
Supplement: Supplementary file 1 [file pharmaceutics-14-02227-s001.zip › pharmaceutics-1947855-supplementary.pdf]

pharmaceutics

Supplementary Materials

Molecular Imaging of Ultrasound-Mediated Blood-Brain Barrier Disruption in a Mouse Orthotopic Glioblastoma Model

Chiara Bastiancich [1,†], Samantha Fernandez [2,†], Florian Correard [3], Anthony Novell [4], Benoit Larrat [5], Benjamin Guillet [2,6,*,‡] and Marie-Anne Estève [3,*,‡]

1 CNRS, INP, Inst Neurophysiopathol, Aix-Marseille University, 13005 Marseille, France
2 Centre Européen de Recherche en Imagerie Médicale (CERIMED), CNRS, Aix-Marseille University, 13005 Marseille, France
3 APHM, CNRS, INP, Inst Neurophysiopathol, Hôpital Timone, Service Pharmacie, Aix Marseille University, 13005 Marseille, France
4 CEA, CNRS, Inserm, BioMaps, Service Hospitalier Frédéric Joliot, Université Paris-Saclay, 91401 Orsay, France
5 CEA, CNRS, NeuroSpin/BAOBAB, Université Paris-Saclay, 91191 Gif-sur-Yvette, France
6 Centre de Recherche en Cardiovasculaire et Nutrition (C2VN), INSERM, INRAE, Aix-Marseille University, 13005 Marseille, France
* Correspondence: benjamin.guillet@univ-amu.fr (B.G.); marie-anne.esteve@univ-amu.fr (M.-A.E.)
† These authors contributed equally to this work.
‡ These authors contributed equally to this work.

Supplementary data

Section S1. BBB permeabilization and [99mTc]Tc-DTPA SPECT/CT imaging on healthy and tumor-bearing animals: raw data.

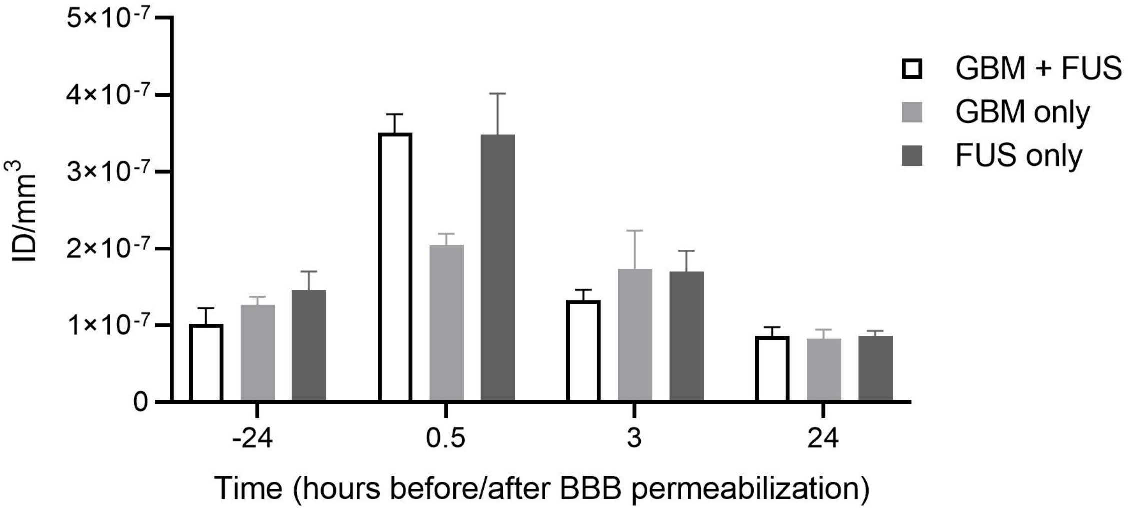

Figure S1. Quantification of [99mTc]Tc-DTPA activity in animals of group "GBM+FUS" (tumor-bearing animals undergoing FUS-mediated BBB permeabilization, $n = 5$), group "GBM only" (tumor-bearing animals without FUS-mediated BBB permeabilization, $n = 5$) and group "FUS only" (healthy animals undergoing FUS-mediated BBB permeabilization, $n = 4$). Results are expressed as injected dose per tissue volume (ID/mm^3; mean ± SEM).

Section S2. BBB permeabilization and [18F]FDG PET/CT imaging on healthy and tumor-bearing animals: raw data.

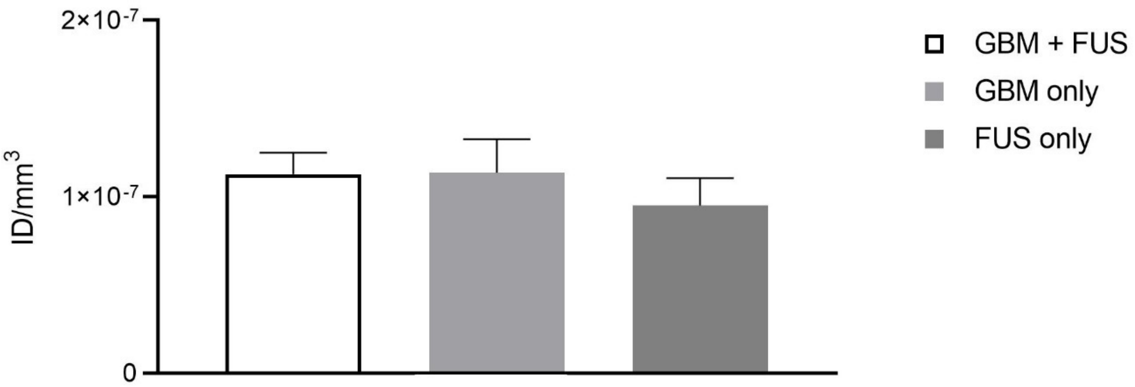

Figure S2. Quantification of [18F]FDG activity in animals of group "GBM+FUS" (tumor-bearing animals undergoing FUS-mediated BBB permeabilization, n = 5), group "GBM only" (tumor-bearing animals without FUS-mediated BBB permeabilization, n = 5) and group "FUS only" (healthy animals undergoing hemispheric FUS-mediated BBB permeabilization, n = 4). Results are expressed as injected dose per tissue volume (ID/mm^3; mean ± SEM).
